# Protective effect of S-nitrosoglutathione pretreatment on acute lung injury in septic rats 

**DOI:** 10.22038/ijbms.2020.43590.10240

**Published:** 2020-08

**Authors:** Zhou-feng Wang, Yu-min Yang, Heng Fan

**Affiliations:** 1 Department of Respiratory and Critical Care, Ningbo First Hospital Longshan Hospital Medical and Health Group, Ningbo, Zhejiang Province, P.R China; 2 Department of Intensive Care Unit, Ningbo First Hospital, Ningbo, Zhejiang Province, P.R China

**Keywords:** Acute lung injury, NF-Κb, Oxidative stress, Sepsis, S-nitrosoglutathione, TLR4

## Abstract

**Objective(s):**

To investigate the protective effect of S-nitrosoglutathione (SNG) pretreatment on acute lung injury (ALI) in septic rats.

**Materials and Methods:**

We constructed a rat model of sepsis by cecal ligation and perforation (CLP), and randomly divided into Sham, CLP, and CLP+SNG (0.25 and 0.5 mg/kg) groups. We used H&E staining and lung wet/dry ratio to assess the severity of lung injury, detected the levels of protein and cells in bronchoalveolar lavage fluid (BALF) and the levels of TNF-α, IL-1β, TLR4 mRNA, and NF-κB p65 mRNA in the lung tissue, and assessed the levels of glutathione (GSH), superoxide dismutase (SOD), glutathione peroxidase (GSH-Px), and catalase in the lung tissue.

**Results:**

A rat model of sepsis was successfully constructed by CLP, and pretreatment of SNG significantly increased the survival of septic rats (*P*<0.001) and decreased the lung tissue injury scores (*P*<0.001) and lung wet/dry ratio (*P*<0.01) in a dose-dependent manner. Furtherly, SNG pretreatment significantly reduced the number of total cells, total protein, neutrophils, and lympholytes (all *P*<0.001) in BALF, and which also decreased the levels of TNF-α, IL-1β, TLR4 mRNA, and NF-κB p65 mRNA (all *P*<0.001) in the lungs of CLP-induced rats. Moreover, pretreatment of SNG significantly increased the levels of anti-oxidant enzymes GSH, SOD, GSH-Px, and catalase (all *P*<0.001) in the lung tissue of septic rats.

**Conclusion:**

SNG pretreatment has a protective effect on ALI in septic rats, and the specific mechanism may be related to anti-endotoxic, anti-inflammatory, and anti-oxidative properties.

## Introduction

Acute lung injury (ALI) is a critical illness in the intensive care unit (ICU), and the incidence continues to rise, eventually leading to acute respiratory distress syndrome (ARDS) (1). According to statistics, the incidence of ALI in septic patients is as high as 40–65%, and the mortality rate is more than 45%, which is one of the important causes of death in ICU patients (2, 3). The main features of patients with septic ALI are extensive inflammation of the lungs, persistent respiratory distress, and refractory hypoxemia. CLP-induced sepsis can trigger the toll-like receptor 4 (TLR4), activate the NF-κB pathway, and produce large amounts of inflammatory factors, such as IL-1, TNF-α, and IL-6 (4). Activation of the inflammatory signaling pathway and mass production and release of cytokines are important pathophysiological mechanisms leading to septic ALI (5). Exploring the pathogenesis of ALI caused by sepsis and finding new therapeutic drugs is an urgent problem to be solved. 

S-nitrosoglutathione (SNG) is an s-nitrosothiol synthesized from intracellular thiols and glutathione (GSH), and which is abundant in human cells and chemically stable (6). Our previous experiments confirmed that SNG has a significant protective effect on septic mice, and its specific mechanism is related to the regulation of the TLR4-NF-κB signaling pathway (7). Gaston *et al*. (8) found that endogenous SNG is active in human cells, and protects normal cell function by stabilizing nitric oxide biological activity and reducing cytotoxicity. Moreover, Langford *et al*. (9) showed that exogenous SNG also has active biological characteristics, and can effectively inhibit platelet aggregation and adhesion in arterial vessels after coronary intervention. 

In the present study, to investigate the preventive and protective effects of SNG pretreatment on ALI in septic rats, we constructed a rat model of sepsis by cecal ligation and perforation (CLP) to observe the effect of SNG pretreatment on the survival of rats. Furtherly, we used a variety of molecular biology experiments to clarify the specific mechanism of SNG pretreatment on protecting ALI in septic rats and to elucidate its important anti-inflammatory and anti-oxidative role. 

## Materials and Methods

***Animals and grouping***

Male Sprague-Dawley (SD) rats (n=120), 14±2 weeks old, weighing 150±20 g, were purchased from Zhejiang University and were raised in the Experimental Animal Center of Ningbo University. Rats were housed at 22±1 °h room temperature, 55–65% humidity, light and dark time for 12 hr each, fasting, and no drinking water 8 hr before CLP. We randomly divided the rats into 4 groups: Sham, CLP, CLP+SNG (0.25 mg/kg, Sigma-Aldrich Co. LLC, Shanghai, China), and CLP+SNG (0.5 mg/kg), and the drug doses were selected based on our previous results of the study(7). We used the CLP method to construct a rat model of sepsis according to our previous experimental method: intraperitoneal injection of sodium pentobarbital (10 mg/kg), a 2–3 cm incision along the midline of the abdomen, locate the end of the cecum, penetrate the cecum with 20^#^ needle, close after proximal ligation of the abdominal cavity (7). The surgical procedure of the Sham group was the same as that of the CLP group, but the cecum was not punctured and ligated. In the CLP+SNG group, rats were given SNG (0.25 mg/kg and 0.5 mg/kg) intragastrically before CLP, once a day for 5 consecutive days, and a model of sepsis was constructed by CLP, 3 hr after the last administration. All animal experiments were performed in accordance with the Laboratory Animal Operating Guidelines and approved by the Experimental Animal Ethics Committee of the Ningbo University (AEWC-2017-33).

***Survival study***

To investigate the effect of SNG on the survival of septic rats, we randomly divided 80 rats into 4 groups: Sham, CLP, CLP+SNG (0.25 mg/kg), and CLP+SNG (0.5 mg/kg), 20 in each group. Rats were reared according to experimental animal feeding standards. The survival of the rats was observed every 12 hr for 7 days. 

***Detection of the*** ***protein and cells*** ***in bronchoalveolar lavage fluid (BALF)***

The other 40 rats were randomly divided into 4 groups (the same as the experimental group above). Twenty-four hours after CLP-induced sepsis, the rats were sacrificed. We opened the chest cavity and removed the left lung for measuring the wet/dry ratio, and then we ligated the middle of the right lung with a thin thread and removed the lower lobe of the right lung for subsequent experiments. We dissected the neck, exposed the trachea, inserted the indwelling needle, fixed with a thin ligature, pulled the needle core, linked the syringe, and the upper lobe of the right lung was lavaged with PBS (0.5 ml) to obtain BALF. The supernatant was obtained by centrifugation at 1500 rpm for 5 min, and the total protein in the BALF supernatant was detected according to the manufacturer’s instructions. After resuspending the pelleted cells in 0.5 ml of PBS, the cells were counted using a hemocytometer.

***H***&***E staining***

Part of the right lung was fixed in 4% paraformaldehyde for 24 hr, embedded in paraffin, sliced by a microtome, and the thickness was 2–3 μm. We stained sections with hematoxylin and eosin (H&E). Pathological damage of the lung tissue was observed under a light microscope by two pathologists, and three fields were randomly observed in each slice. We performed semi-quantitative scoring by the severity of pulmonary edema, hemorrhage, and inflammatory cell infiltration, and divided it into 0-4 grades: 0, no damage; 1, mild; 2, moderate; 3, severe; 4, very severe.

***Lung wet/dry ratio determination***

Twenty-four hours after construction of septic rats, the rats were sacrificed and their left lungs were taken for evaluation of the wet/dry ratio of the lungs. The fresh lungs were first weighed by an electronic scale, and then the lungs were dried in a 70 °r oven for 48–76 hr until the weight was constant. The electronic scale weighed the dry weight of the lungs and calculated the ratio by the wet/dry formula. 

***ELISA***

Part of the right lung was crushed by a grinder, homogenized in 1 ml of PBS, allowed to stand for 30 min, centrifuged at 1000 rpm for 3 min, and the supernatant was taken. We used ELISA to detect TNF-α and IL-1β levels in the lung tissue according to the manufacturer’s instructions (Invitrogen co., Shanghai, China). 

***RT-qPCR***

A small amount of fresh lung tissue was ground using a tissue grinder (TissueLyser II, Shanghai, China). We performed total RNA purification according to the instructions (RNeasy Mini Kit, Qiagen, Germany) and generated complementary DNA by reverse transcription. We used RT-qPCR system (StepOne Plus, Shanghai, China) to analyze the relative expression of TLR4 mRNA and NF-κB p65 mRNA, and used the following primers: TLR4, Forward primer, 5’-CAA CTG CGC CAC CCC CAA CAT ACT GTC-3’; Reverse primer, 5’- CGC CTC CTA CTT CTA CCA CGG CAT CGC-3’; NF-κB p65, Forward primer, 5’-CAT CAA CGC CGG CAT CCC CGC CTC CAT-3’; Reverse primer, 5’ -CGC CCA GTC CGT TTC CAT CGC GCC-3’. Specific parameters: 50 ^°^C 2 min, 95 ^°^C 10 min, 94 ^°^C 60 sec, 60 ^°^C 60 sec, a total of 35 cycles. All data were expressed as relative GAPDH expression levels, and a 2^-ΔΔCt^ value was calculated.

***Detection of anti-oxidant levels***

We measured the level of glutathione (GSH) in the lung tissue according to the method of Ye *et al*. (10) and evaluated the activity of superoxide dismutase (SOD) by measuring the absorbance at 560 nm. We assessed the glutathione peroxidase (GSH-Px) level of the lung tissue by absorbance at 340 nm according to the method provided by Baydas *et al*. (11) and determined the level of catalase by measuring the absorbance at 420 nm.

***Statistical methods***

We used the GraphPad Prism 6.25 (San Diego, CA, US) software package to map and perform statistical analysis. We expressed all data as mean±standard deviation, and performed survival analysis using a log-rank Mantel-Cox test, used oneway-ANOVA followed by the Tukey-Kramer test for comparison between groups. *P*<0.05 was considered to be significant. 

## Results

***Protective effect of SNG pretreatment on ALI induced by CLP***

**Figure 1:**
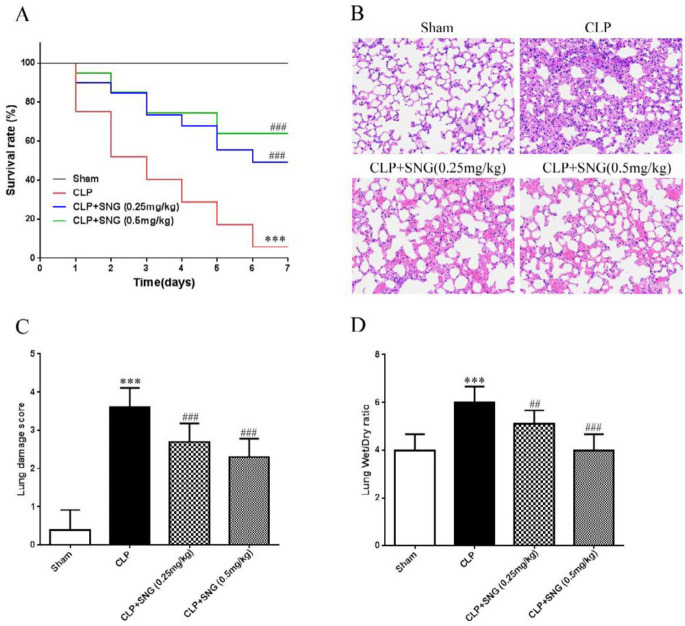
Protective effect of s-nitrosoglutathione pretreatment on septic rats induced by cecal ligation and perforation. (A) effect of s-nitrosoglutathione pretreatment on cecal ligation and perforation-induced mortality (20 rats in each group); (B) pathological changes of the lung tissue in four groups of rats; (C) pathological score of the lung tissues; (D) assessment of pulmonary edema by measuring lung wet/dry ratio. Data were presented as means±SEM, and 10 rats in each group. ***P<0.001 vs Sham group; ##*P*<0.01, ###*P*<0.001 vs CLP group. SNG: s-nitrosoglutathione; CLP: cecal ligation and perforation

We successfully constructed a rat model of sepsis by CLP and monitored the survival of the rats every 12 hr for 7 days. Rats had a survival rate of 100% within 7 days and behavior was normal in the Sham group. The mortality of rats was as high as 95% within 7 days in the CLP group (*P*<0.001), while the mortality of rats in CLP+SNG (0.25 mg/kg) and CLP+SNG (0.5 mg/kg) pretreatment groups decreased to 45% (*P*<0.001) and 35% (*P*<0.001), respectively (Figure 1A). Then we assessed the pathological changes in the lung tissue using H&E staining. In the CLP group, alveolar and interstitial edema was observed, the alveolar wall thickened, and a large number of inflammatory cells infiltrated in the interstitium. In contrast, pathological lesions in the lung tissues of SNG (0.25 mg/kg) and SNG (0.5 mg/kg) pretreatment groups were significantly alleviated, especially alveolar wall thickening and inflammatory cell infiltration were observed (Figure 1B). Compared with the CLP group, the lung tissue injury scores of SNG (0.25 mg/kg) (*P*<0.001) and SNG (0.5 mg/kg) (*P*<0.001) pretreatment groups were significantly reduced in a dose-dependent manner (Figure 1C). In addition, CLP resulted in a significant increase in the lung wet/dry ratio (*P*<0.001), whereas SNG pretreatment significantly reduced this ratio (*P*<0.01) (Figure 1D). 

***SNG pretreatment improves inflammatory infiltration in ALI in septic rats***

**Figure 2.**
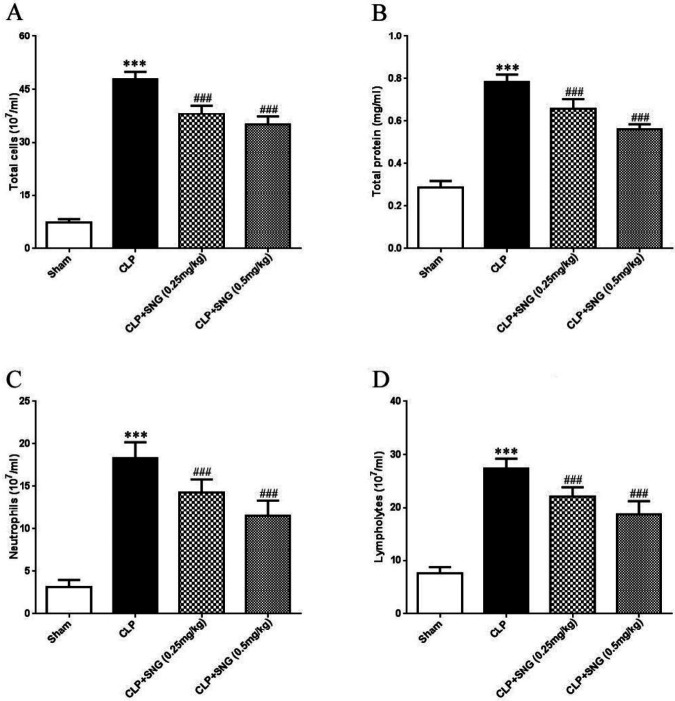
Effect of s-nitrosoglutathione pretreatment on total cell, total protein, lymphocyte and neutrophil counts in bronchoalveolar lavage fluid of rats induced by cecal ligation and perforation. (A) total number of cells in bronchoalveolar lavage fluid; (B) total protein in bronchoalveolar lavage fluid; (C) lymphocyte counts in bronchoalveolar lavage fluid; (D) neutrophil counts in bronchoalveolar lavage fluid. Data were presented as means±SEM, and 10 rats in each group. ***P<0.001 vs Sham group; ###*P*<0.001 vs CLP group. SNG: s-nitrosoglutathione; BALF: bronchoalveolar lavage fluid; CLP: cecal ligation and perforation

To investigate the therapeutic effects of SNG pretreatment on lung inflammation in septic rats, we examined the number of total cells, total protein, neutrophils, and lympholytes in BALF. The number of inflammatory cells was at a low level in the BALF of the sham group, while the numbers of total cells, total protein, neutrophils, and lympholytes were significantly increased in the BALF of the CLP group, and the difference was significant between the two groups (all *P*<0.001) (Figures 2A-D). However, SNG pretreatment significantly reduced the number of total cells, total protein, neutrophils, and lympholytes in BALF of CLP-induced rats in a dose-dependent manner (all *P*<0.001) (Figures 2A-D). These results confirmed that SNG pretreatment inhibited lung inflammation and had significant protective effects in septic rats. 

***SNG pretreatment inhibits inflammatory reaction in the lung tissue of rats induced by CLP***

**Figure 3:**
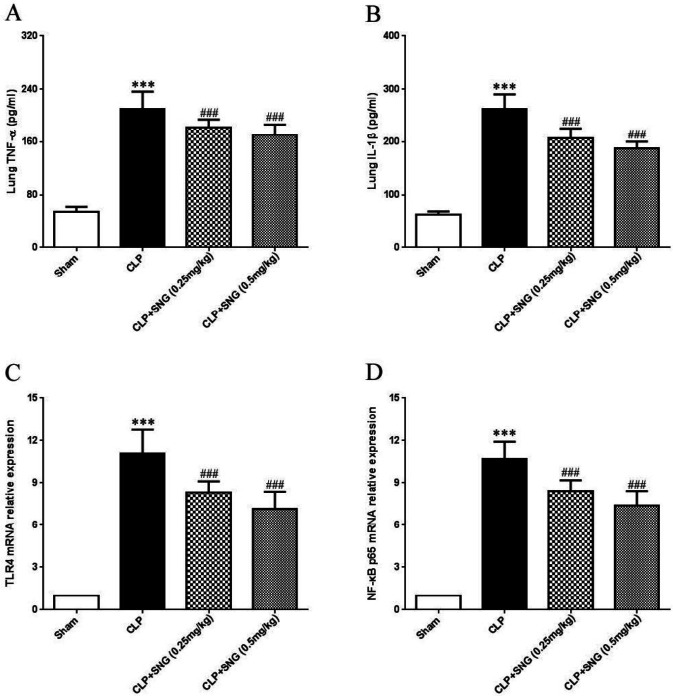
Inhibitory effect of s-nitrosoglutathione pretreatment on inflammatory reaction in the lung tissue of septic rats. (A) level of TNF-α in the lung tissue by enzyme linked immunosorbent assay; (B) level of IL-1β in the lung tissue by ELISA; (C) level of toll-like receptor4 mRNA by RT-qPCR; (D) level of NF-κB p65 mRNA by RT-qPCR. The expression of TLR4 and NF-κB p65 mRNA in the lungs was normalized to GAPDH. Data were presented as means±SEM, and 10 rats in each group. ***P<0.001 vs Sham group; ###*P*<0.001 vs CLP group. SNG: s-nitrosoglutathione; TLR4: toll-like receptor4; CLP: cecal ligation and perforation

To explore the anti-inflammatory effect of SNG pretreatment on ALI in septic rats, ELISA was used to detect TNF-α and IL-1β levels in the lung tissue. We found that TNF-α and IL-1β levels were significantly elevated in the lung tissue of the CLP group (all *P*<0.001), while SNG pretreatment significantly reduced TNF-α and IL-1β levels (all *P*<0.001) (Figures 3A, B). Furtherly, we examined the expression levels of TLR 4 and NF-κB p65 mRNA in the lung tissue by RT-qPCR. We found that the expression levels of TLR 4 and NF-κB p65 mRNA were significantly increased in the lungs of CLP-induced rats (all *P*<0.001), while SNG pretreatment significantly reduced the expression of TLR4 and NF-κB p65 mRNA in the lungs (all *P*<0.001), and 0.5 mg/kg SNG reduced TLR4 and NF-κB p65 mRNA in the lungs by 1.2–1.5 and 1.1–1.3 fold, respectively (Figures 3C, D). Our results suggested that SNG pretreatment significantly inhibited the activation of the TLR4/NF-κB signaling pathway in the lung tissue of septic rats.

***SNG pretreatment attenuates oxidative stress of ALI in septic rats***

**Figure 4:**
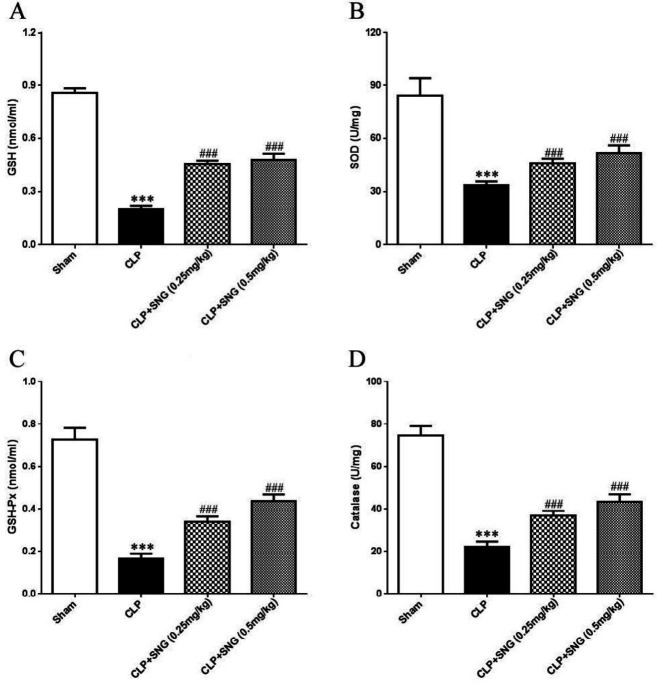
Effect of s-nitrosoglutathione pretreatment on oxidative stress in the lung tissue of septic rats. (A) level of lung glutathione; (B) level of lung superoxide dismutase; (C) level of lung glutathione peroxidase; (D) level of lung catalase. Data were presented as means ± SEM, and 10 rats in each group. ***P<0.001 vs Sham group; ###*P*<0.001 vs CLP group. SNG: s-nitrosoglutathione; GSH: glutathione; SOD: superoxide dismutase; GSH-Px: glutathione peroxidase; CLP: cecal ligation and perforation

To determine the anti-oxidant effect of SNG pretreatment on ALI in septic rats, we examined the levels of anti-oxidant enzymes GSH, SOD, GSH-Px, and catalase in the lung tissue. Compared with the Sham group, GSH levels decreased by 62.5% in the CLP group, while SNG (0.25 mg/kg) and SNG (0.5 mg/kg) pretreatment inhibited CLP-induced GSH decline by 40.1% and 37.7%, respectively (all *P*<0.001) (Figure 4A). SOD levels decreased by 48.1% in the CLP group, and SNG (0.25 mg/kg) and SNG (0.5 mg/kg) pretreatment inhibited GSH decline by 43.7% and 32.9%, respectively (all *P*<0.001) (Figure 4B). For another anti-oxidant enzyme, GSH-Px activity was reduced by 57.1% in the CLP group, and SNG (0.25 mg/kg) and SNG (0.5 mg/kg) pretreatment inhibited 38.2% and 34.4% of the decrease in CLP-induced GSH-Px activity, respectively (all *P*<0.001) (Figure 4C). Similarly, the level of catalase in the CLP group was 55.4% lower than that in the Sham group, while the pretreatment of SNG (0.25 mg/kg) and SNG (0.5 mg/kg) inhibited 39.7% and 37.2% of the catalase levels in the CLP group, respectively (all *P*<0.001) (Figure 4D). Our results suggested that SNG pretreatment inhibited CLP-induced oxidative stress in the lung tissue of rats, and had an anti-oxidant function. 

## Discussion

Sepsis is a common critical illness in ICU and one of the common causes of ALI/ARDS, with high morbidity and mortality(12). Early prevention of sepsis development can effectively reduce the incidence of ALI/ARDS and reduce the mortality of patients. Studies showed that ALI/ARDS caused by sepsis is the result of uncontrolled inflammatory response in the body (13). In this study, we successfully constructed a rat model of sepsis by CLP. We found that SNG pretreatment reduced the mortality of septic rats, and had significant protective effects. Furthermore, we observed pathological damage of the lung tissue in septic rats. We found that SNG pretreatment attenuated the severity of pathological damage in the lung tissue and reduced the pathological damage score and wet/dry ratio. These results led us to further explore the specific molecular biological mechanisms of SNG in ALI protection in septic rats. 

The inflammatory reaction is a powerful counterattack against the invasion, and the immune cells are called to reach the injury site in a few minutes. Inflammatory factors pass through the vascular endothelium, and white blood cells release a large amount of lytic enzymes that penetrate into the basement membrane composed of the extracellular matrix, causing damage to the integrity of the lung tissue (14). Previous studies on ALI mainly focused on inflammatory responses and cell damage. In recent years, the self-repair function of the lung has gradually gained attention, such as dissipation of edema and inflammation, cell proliferation, and tissue remodeling (15). The repair effect may be initiated at the beginning of the injury and plays an important role in the later recovery phase. Therefore, early antagonism of the inflammatory response to prevent damage may greatly hinder the post-repair process. 

The direct response of the body to infection and trauma is the release of inflammatory mediators, mainly including TNF-α, IL-1β, and IL-6 (16). TNF-α and IL-1β levels are generally considered to be initiators of inflammation, and IL-6 level is a marker of cytokine cascade activation and prognosis of inflammatory responses. SNG treatment may ameliorate intestinal and lung injury in rats by inhibiting inflammatory response and oxidative stress (17). TLR4 as an important member of the toll-like receptor family is a transmembrane receptor in the immune system and activates downstream signal transduction molecules by recognizing pathogen-associated molecular patterns (18). The TLR4 pathway is also a classical pathway for inducing inflammatory signaling in cells and plays an important role in the activation of inflammatory reactions and immunomodulation in sepsis (19). TLR4 recognizes pathogenic model molecules and activates NF-κB through a variety of pathways, and binds to TNF-α and IL-1β promoter genes to promote cascade amplification of inflammatory responses (20). 

To investigate the therapeutic effects of SNG pretreatment on lung inflammation in septic rats, we assessed inflammatory infiltration in the lung tissue. We found that SNG pretreatment significantly reduced the number of total cells, total protein, neutrophils, and lympholytes in BALF of CLP-induced rats in a dose-dependent manner. Moreover, we determined the degree of ALI inflammatory response by detecting levels of the lung tissue inflammatory factors TNF-α, IL-1, TLR4 mRNA, and NF-κB mRNA. Our results showed that the levels of TNF-α, IL-1, TLR4 mRNA, and NF-κB mRNA in the lung tissue of the CLP group were significantly increased compared with the sham group, suggesting that the CLP method was successfully used to prepare the ALI model of septic rats. For the SNG pretreatment group, the levels of TNF-α, IL-1, TLR4 mRNA, and NF-κB mRNA in the lung tissue decreased significantly, indicating that SNG pretreatment reduced the inflammatory response of septic ALI, and the specific mechanism might be related to blocking the TLR4/NF-κB signaling pathway. 

GSH is composed of glutamic acid, cysteine, and glycine, and can promote the repair of immune system function and has anti-oxidation and detoxification effects (21). SOD is an important anti-oxidant enzyme in the body, and can alleviate lung damage caused by oxygen-free radicals. GSH-Px is an important oxygen-free radical scavenging enzyme, and can eliminate peroxides, free radicals produced, and prevent the lung tissue from oxidative damage (22). Catalase is widely distributed in mammalian cells, and mainly catalyzes the decomposition of H_2_O_2_ into H_2_O and O_2_ (23). In the present study, we found that SNG pretreatment can increase the levels of anti-oxidant enzymes GSH, SOD, GSH-Px, and catalase in the lung tissue, and has significant anti-oxidant and free radical scavenging function.

## Conclusion

The imbalance of pro-inflammatory and anti-inflammatory factors is the main pathophysiological mechanism of CLP-induced ALI. SNG has anti-endotoxic, anti-inflammatory and anti-oxidative pharmacological effects, and can significantly inhibit inflammation in the lung tissue induced by CLP, and the specific mechanism may be achieved by regulating TLR4/NF-κB. Besides, SNG pretreatment can increase the level of anti-oxidant enzymes in the lung tissue and improve the survival rate of septic rats, which has a significant protective effect on ALI in septic rats.
